# Microbial diversity and ecotoxicity of sediments 3 years after the Jiaozhou Bay oil spill

**DOI:** 10.1186/s13568-018-0603-6

**Published:** 2018-05-09

**Authors:** Wei Gao, Xiaofei Yin, Tiezhu Mi, Yiran Zhang, Faxiang Lin, Bin Han, Xilong Zhao, Xiao Luan, Zhisong Cui, Li Zheng

**Affiliations:** 1 College of Marine Life Sciences, Ocean University of China, Qingdao, 266003 China; 2 Key Laboratory for Marine Bioactive Substances and Modern Analytical Technology, The First Institute of Oceanography, State Oceanic Administration of China, No. 6 Xianxialing Road, Qingdao, 266000 Shandong People’s Republic of China; 3 College of Environmental Science and Engineering, Ocean University of China, Qingdao, 266100 China; 4 Key Laboratory of Marine Chemical Theory and Technology, Ministry of Education, Qingdao, 266100 China; 5 Laboratory for Marine Ecology and Environmental Science, Qingdao National Laboratory for Marine Science and Technology, Qingdao, 266071 China

**Keywords:** Oil spill, Petroleum hydrocarbons, Sediment, Bacterial diversity, Biotoxicity

## Abstract

In 2013, the “Qingdao oil pipeline explosion” released an estimated 2000 tons of oil into the environment. Sediment samples were collected from ten sites in Jiaozhou Bay and Shilaoren Beach to evaluate the influence of the spilled oil on the benthic environment 3 years after the oil spill accident. The compositions of oil, bacterial diversity and biotoxicity were examined in this study. The results showed that the concentration of total petroleum hydrocarbons (TPHs) peaked near the oil leak point and gradually decreased along the coastline, ranging from 21.5 to 133.2 μg/g. The distribution of polycyclic aromatic hydrocarbons (PAHs) was correlated with TPH, and naphthalenes were dominant in the 20 detected PAHs. The bacterial diversities in seriously polluted and slightly polluted sediments were completely different. As degrading bacteria, *Alcanivorax* and *Lutibacter* were the main genera at the oil-polluted sites. The analysis of biotoxicity by the luminescent bacteria method showed great differences among the polluted sites, the control site in Jiaozhou Bay, and the non-polluted site outside of Jiaozhou Bay. The biotoxicity also peaked at the site near the oil leak point. These results indicate that the oil spill that occurred 3 years ago still affects the environment and impacts the bacterial communities in the sediments.

**Electronic supplementary material:**

The online version of this article (10.1186/s13568-018-0603-6) contains supplementary material, which is available to authorized users.

## Introduction

On Nov 22nd, 2013, an oil pipeline exploded in Huang Dao, Qingdao City, China. The crude oil spilled into the sea through the municipal pipelines. An estimated 2.5 km of shoreline was seriously polluted by the oil spill. To mitigate the impact of the oil, dispersants were applied to surface waters around the leak point. The use of the dispersants was meant to promote the deposition of the oil to the sea floor (Gong et al. 2014). Meanwhile, the harbour (near the leak point in Jiaozhou Bay-an inner sea) might prevent the oil from reaching the open sea. The special hydrological characteristics of these sites might keep the oil near the shoreline where it could persist for a long time. Therefore, the spilled oil would have a different impact on the benthic environment.

Major concerns were raised about the long-term influence of the spilled oil on the marine environment (Hong et al. 2012; Peterson et al. 2003; White et al. 2014). To assess the ecological impact of the spilled oil, it was important to know the full extent and level of contamination at impacted locations as well as how the oil spill impacted the bacterial community. The responses of bacterial communities to oil spills have frequently been studied, especially in Mexico after the Deepwater Horizon oil spill (Acosta-González et al. 2013; King et al. 2015). Recently, studies began focusing on the long-term impacts of the Deepwater Horizon oil spill on the environment (Yergeau et al. 2015; Beyer et al. 2016; Romero et al. 2017). In this study, we sampled the polluted sediments, identified the diversity of the bacterial community and tested the biotoxicity of the polluted sediments. The samples from the non-contaminated areas served as controls to distinguish the oil spill signature from the background hydrocarbons. This research provides basic information on the long-term influence of oil contamination on the benthic environment.

## Materials and methods

### Sampling and experimental design

**Fig. 1:**
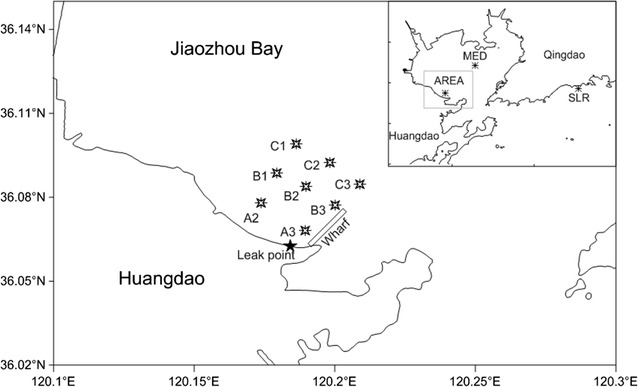
Map of the sediment sampling sites in Jiaozhou Bay. The symbol filled black star identifies the location of the leak point, and  identifies the sampling sites. A, B and C were the three sampling sections and were parallel to the shoreline. The sampling sites were designated with a line name and number, e.g., B1, B2 and B3 were the sites in line B. Eight of the ten sites were located near the leak point. The sites MED and SLR were chosen as control sites on the other side of Jiaozhou Bay and Shilaoren Beach

Sediments were collected using a bottom sampler. We selected three sampling sections (i.e., A, B, and C) parallel to the shoreline. The sampling sites were designated based on the line name and number, e.g., B1, B2 and B3 were the sites in line B. The three sites that were perpendicular to the shoreline of the contaminated site were named A3, B3, and C3. In total, there were ten sites in this study. Eight sites were placed around the leak point. Two sites named as MED and SLR were chosen as control sites on the other side of Jiaozhou Bay and Shilaoren Beach (Fig. 1). The collected samples were wrapped in tinfoil and persevered at 4 °C.

### DNA extraction and sequencing

#### Extraction of genomic DNA from sediment samples

Total genomic DNA was extracted from samples using the CTAB/SDS method (Rochelle 2001). DNA concentration and purity were monitored on 1% agarose gels. According to the concentration, sterile water was used to dilute the DNA to 1 ng/μL. Prepared DNA samples were sent to Novegene (Beijing, China). The five sites were chosen for the microbial community analysis and included the three seriously polluted sites (i.e., A3, B3, C3) and the two control sites (i.e., MED and SLR).

#### Amplification and 16S rRNA gene sequencing

The V3-V4 regions were amplified using Phusion^®^ High-Fidelity PCR Master Mix (New England Biolabs). After purification with the Qiagen gel extraction kit (Qiagen, Germany), sequencing libraries were generated using the TruSeq^®^ DNA PCR-Free sample preparation kit (Illumina, USA) following the manufacturer’s recommendations; finally, the library was sequenced on an Illumina HiSeq2500 platform and 250 bp paired-end reads were generated. All the sequence data were submitted to the GenBank Sequence Read Archive under BioProject accession number PRJNA399274.

### Data analysis

Paired-end reads were merged using FLASH (V1.2.7) to splice overlapping sequences to raw tags (Magoč and Salzberg 2011). The raw tags were performed under specific filtering conditions to obtain the high-quality clean tags according to the QIIME (V1.7.0) quality controlled process (Caporaso et al. 2010; Bokulich et al. 2013). The effective tags were finally obtained after chimera removal (Edgar et al. 2011; Haas et al. 2011). Analyses of the sequences were performed using Uparse software (Uparse v7.0.1001) (Edgar 2013). Sequences with ≥ 97% similarity were assigned to the same OTUs. A representative sequence for each OTU was screened for further annotation with the Green Gene Database (DeSantis et al. 2006) based on RDP3 classifier (Version 2.2) (Wang et al. 2007) algorithm to annotate taxonomic information. To study phylogenetic relationships of different OTUs, and the differences in the dominant species present in the different samples (i.e., groups), multiple sequences alignments were conducted using the MUSCLE software (Version 3.8.31) (Edgar 2004). Subsequent analyses of alpha diversity and beta diversity were all performed based on this normalised data output. Six diversity indices, including observed-species, Chao1, Shannon, Simpson, ACE, and good-coverage, were applied in analysing the complexity of species diversity for each sample. Principal component analysis (PCA) was preceded by the FactoMineR package and ggplot2 package in R software (Version 2.15.3).

### Oil concentration and composition analysis

The sediments were freeze-dried (Christ alpha 1-4LD, Germany) before the oil analysis and 20 g of sediment samples were extracted with a mixture of *n*-hexane and dichloromethane. The total petroleum hydrocarbons were determined by the fluorescence spectrophotometry method according to Massoud et al. (1996) with a minor modification. The alkanes and PAHs were detected using GC–MS in triplicate using the procedures described previously (Gao et al. 2015). A 1-mL sample of the sediment extraction was dehydrated in a column with 2 g of anhydrous Na_2_SO_4_ and filtered using 0.22-μm nylon membrane (JINTENG, Tianjin, China). After evaporation under a stream of nitrogen, the residual was re-dissolved in chromatography-grade *n*-hexane. *N*-tetracosane-D50 and *P*-terphenyl-D14 were set as internal standards (10 μg/mL each). The samples were analysed by a 6890A gas chromatograph (Agilent Technologies) in an HP-5 MS capillary column (30 m × 250 μm i.d., 0.22 μm thickness) and a 5973 mass spectrometer equipped with a quadrupole axis detector. The semi-quantification of hydrocarbons was conducted following the protocols described by Zheng (2012).

### Biotoxicity test by luminous bacteria

The biotoxicity of each sample was determined by the luminescent bacteria *Acinetobacter baylyi* HesenATox (Shanghai HeSen Biotechnology co., Ltd). HgCl_2_ was used as a reference standard toxicant with concentrations from 0.06, 0.08, 0.10, 0.12, and 0.14 mg/L. The bacteria were prepared according to the manufacturer’s instructions. The sediments were freeze-dried, and the biotoxicity was detected following the method of Xu (2016). The luminescence intensity was measured using a microplate reader (VictorX PerkinElmer). The toxicity analysis was conducted similar to Luan (2015). The luminous inhibition rate was calculated using the following formula.

$${\text{IHR}} = \left( {{\text{L}}_{\text{CK}} - {\text{Ls}}} \right)/{\text{L}}_{\text{CK}} \times 100\%$$

L_CK_ is the luminous intensity of the negative control and Ls is the luminous intensity of the sample or positive control (HgCl_2_). The linear regression equation was established with the IHR and the corresponding concentration of mercury chloride. The equivalent concentration of the sample was calculated using this regression equation, and the toxicity evaluation referred to Luan’s grading method.

## Results

### Determination of total petroleum hydrocarbons by fluorescence spectrometry

**Fig. 2:**
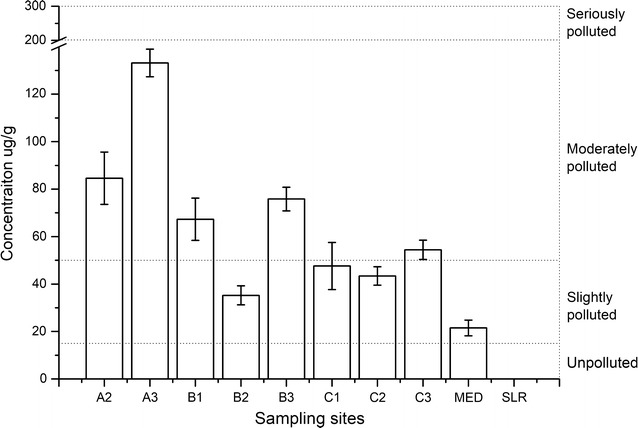
The total petroleum hydrocarbons in sampling sediment analysis by fluorescence spectrophotometry. The concentrations of the TPHs were calculated by the standard curve. The site A3 was next to the leak point. No TPH was detected at site SLR. The symbol An (Bn, Cn): the nst sampling site of line A (B, C); MED: control site in Jiaozhou Bay; SLR: control site in Shilaoren Beach, which was outside of Jiaozhou Bay

The total petroleum hydrocarbons were quantified in this research by fluorescence spectrophotometry. Sediments from ten sites were collected; these included eight sites near the site of the oil spill (i.e., samples A2–C3) and two control sites in Jiaozhou Bay and Shilaoren Beach. The TPH concentrations ranged from 21.5 to 133.17 μg/g. No TPH concentration was detected at the control site SLR. The highest TPH concentration was in the sample from site A3. The concentrations in line A were the highest among the concentrations in the three lines (i.e., lines A, B, and C). The TPH concentrations from sites A3, B3 and C3 were the highest in each corresponding line, as shown in Fig. 2. The TPH concentration was highest in the site near the leak point and gradually decreased along the coastline, both in parallel and perpendicular directions from the leak point. According to the standard pollution levels in bottom sediments (Massoud et al. 1996), the sediments from sites B2, C1, C2, and MED were slightly polluted. The other sites (except for Shilaoren Beach) were moderately polluted. The high concentration found at site MED means oil pollution occurred; slower degradation and weathering rates may have caused oil to accumulate at this site.

### The characteristics of petroleum hydrocarbons in sediments

**Fig. 3:**
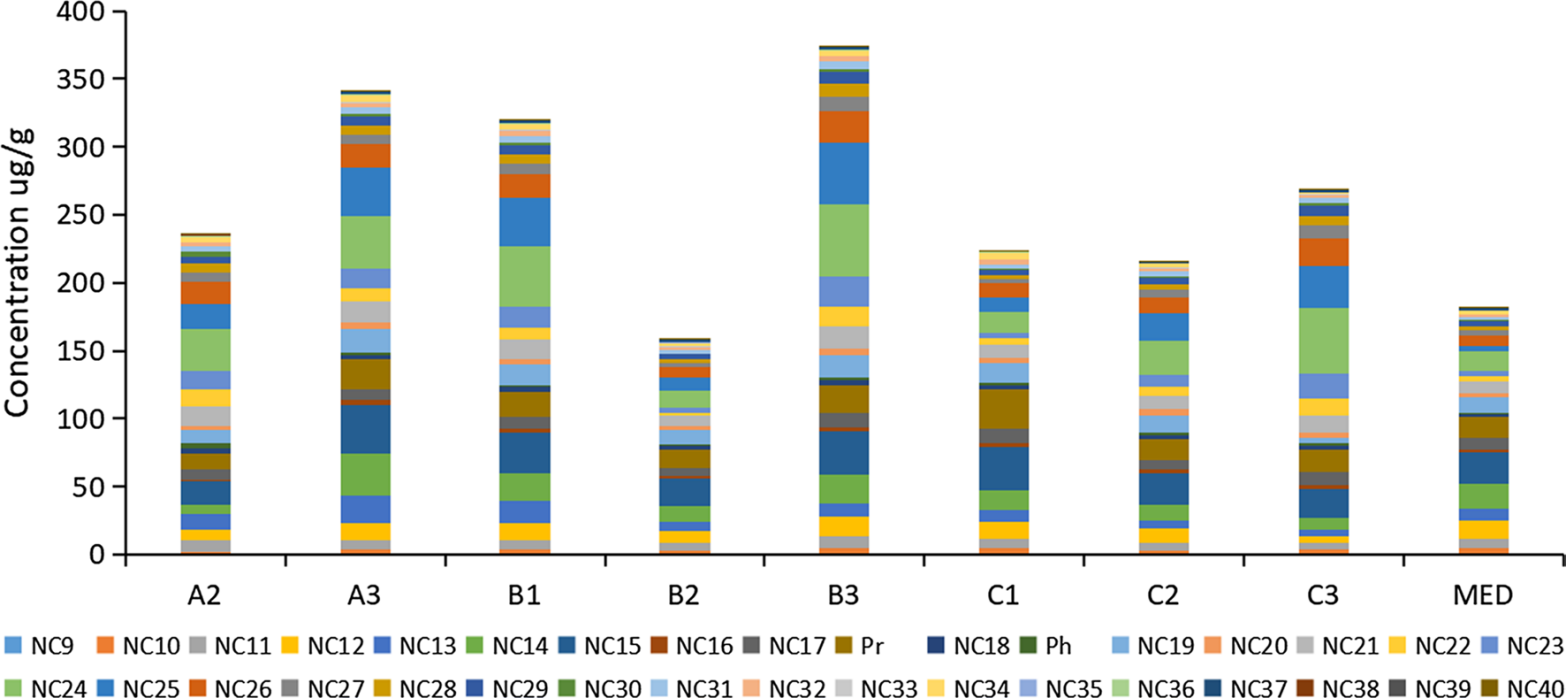
The abundance of *n*-alkanes in sampling sediment analysis by GC–MS. The symbol An (Bn, Cn): the nst sampling site of line A (B, C); MED: control site in Jiaozhou Bay; SLR: control site in Shilaoren Beach, which was outside of Jiaozhou Bay. Internal standards were determined using *n*-tetracosane-D50 (10 μg/mL). The abundance of each n-alkane was calculated by the internal standards

The n-alkane was analysed by the GC–MS method. Samples from nine sites in Jiaozhou Bay were tested. The total alkane concentrations ranged from 157.92 to 373.48 μg/g. Sites A3, B3 and C3 also contained the highest concentrations in each of their corresponding lines. This was consisted with the distribution of TPH concentrations. The concentrations of alkanes in samples A2, B2, C1 and C2 were close to the alkane concentration at the control site MED, while concentrations at the other sites were much higher. In Fig. 3, the abundance of short-chain alkanes (C9–C20) were similar in sites A3, B3, and C3. The most abundant types of carbon in the three sites were middle-chain alkanes (C21–C26). The alkanes with more than 31 carbon atoms were not as abundant. The abundance of each alkane at site MED was quite different from those of the three sites (i.e., A3, B3 and C3); specifically, the abundance of C22–C31 alkanes were much higher in the three sites than in the control site MED.

**Fig. 4:**
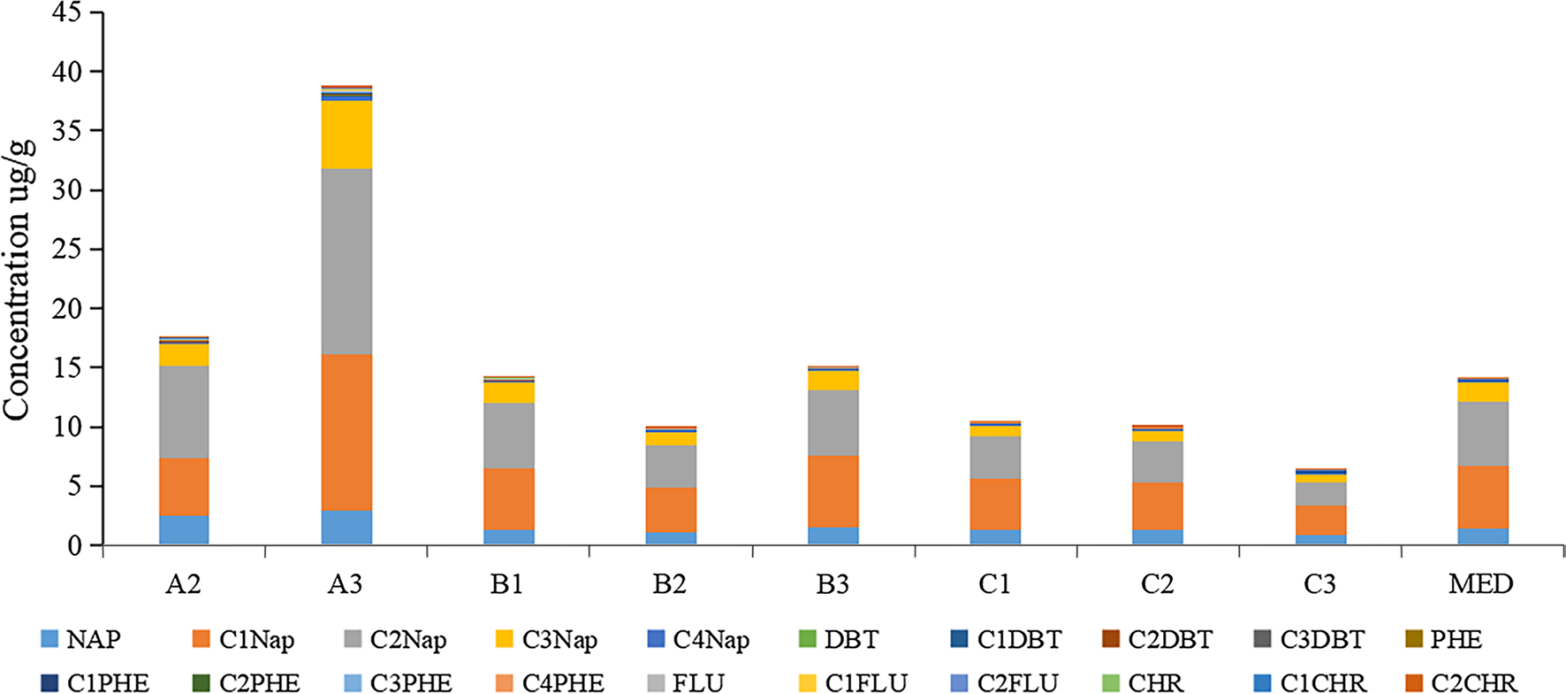
The abundance of PAHs in sampling sediment analysis by GC–MS. The symbol An (Bn, Cn): the nst sampling site of line A (B, C); MED: control site in Jiaozhou Bay; SLR: control site in Shilaoren Beach, which was outside of Jiaozhou Bay. Internal standards were determined using *p*-terphenyl-D14 (10 μg/mL). The abundance of each PAH was calculated by the internal standards

The distributions of PAHs were quite different from the distributions of n-alkanes in the sediments. The abundance of PAHs in site A3 (38.7 μg/g) was the highest of all surveyed sites. The concentration in other sites ranged from 6.4 to 17.5 μg/g. The control site MED was also polluted with PAHs, with a concentration of 14.04 μg/g. The concentration of PAHs in line A was the highest among the three lines (i.e., line A, line B and line C). This was similar to the distributions of n-alkanes. The concentrations of PAHs in lines B and C were similar to the control site. The characteristics of the PAHs were analysed by GC–MS. NAPs was the main component in all the sites (shown in Fig. 4). The sediment samples from site A3 contained the highest concentrations of NAPs and were 52.7–74.9% higher than the control site MED. The concentrations of NAPs in the control site were 64.3–165.0% higher than the concentrations in site C3 (excluding C4-NAP), and site B3 was similar to the control site. This means that site MED in Jiaozhou Bay was contaminated by PAHs to some extent.

### Distribution and diversity of microbial communities in sediments

**Fig. 5:**
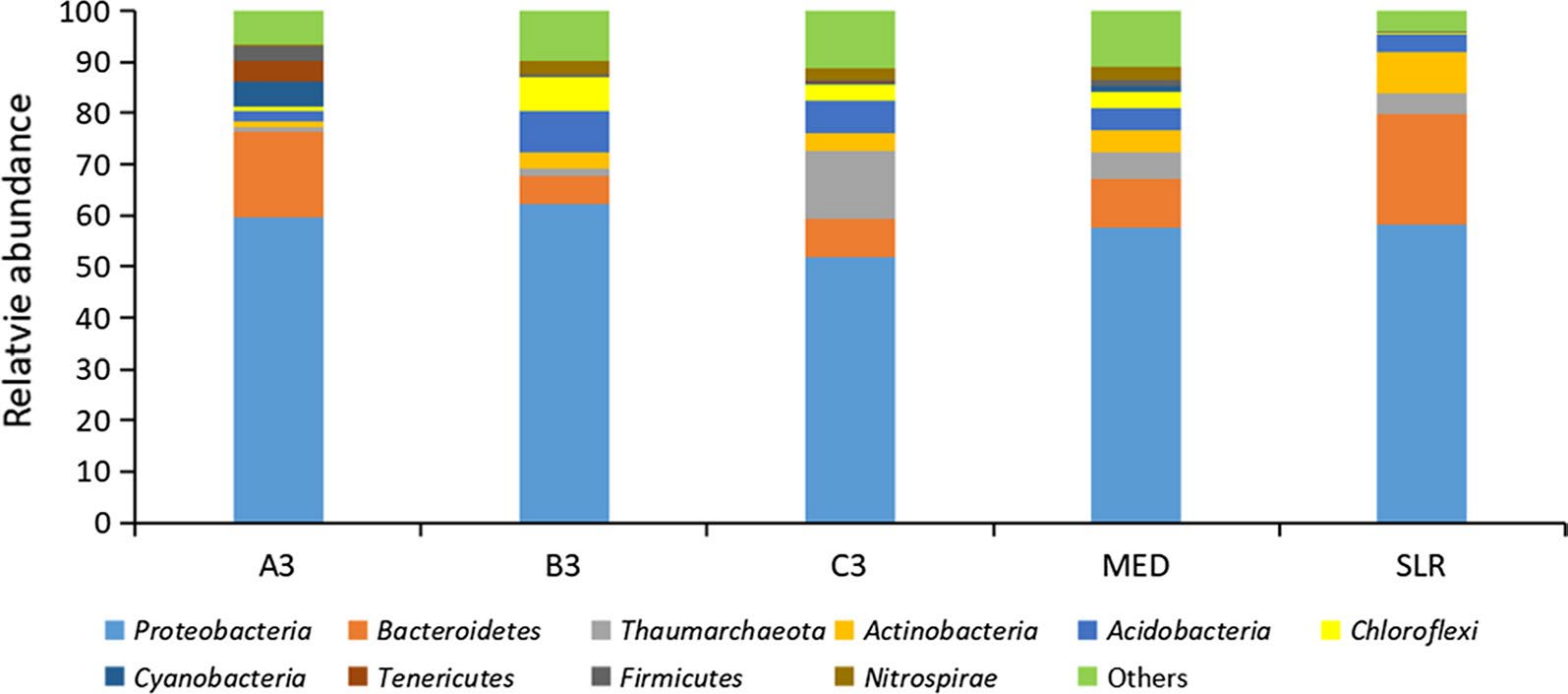
The bacterial biodiversity in the sediments in the direction perpendicular to the shoreline. The map was generated from the highest abundance of the top 10 species at the phylum level. The symbol A3 (B3, C3): the sampling site near the leak point; MED: control site in Jiaozhou Bay; SLR: control site outside of Jiaozhou Bay

The sites A3, B3 and C3, which had higher oil concentrations, were chosen for the study of microbial diversities; in contrast, sites MED and SLR served as control sites (Fig. 5). In all sites, *Proteobacteria* was the main phylum, accounting for 51.97–62.28% of the community. All the sites contained a certain degree of *Thaumarchaeota*, which was different than reports from former studies (e.g., King et al. 2015; Rivers et al. 2013). When comparing the seriously contaminated site (A3) and the other four sites, we found that the abundances of *Actinobacteria* and *Acidobacteria* were much lower at site A3. The abundance of *Bacteroidetes* at site A3 was higher than the abundances at the other three sites (except site SLR). There was also a considerably high abundance of *Bacteroidetes* at site SLR, and this was close to the abundance of the same phylum at site A3. What is interesting is the distribution of the phylum *Tenericutes*. The phylum *Tenericutes* showed a high abundance at site A3 (4.17%). In addition, the compositions of *Tenericutes* at the other four sites were much lower (< 0.1%). Thus, *Tenericutes* was studied as apparent bacteria found in oil-contaminated areas, which meant that the community at site A3 was still affected by the spilled oil.

**Fig. 6:**
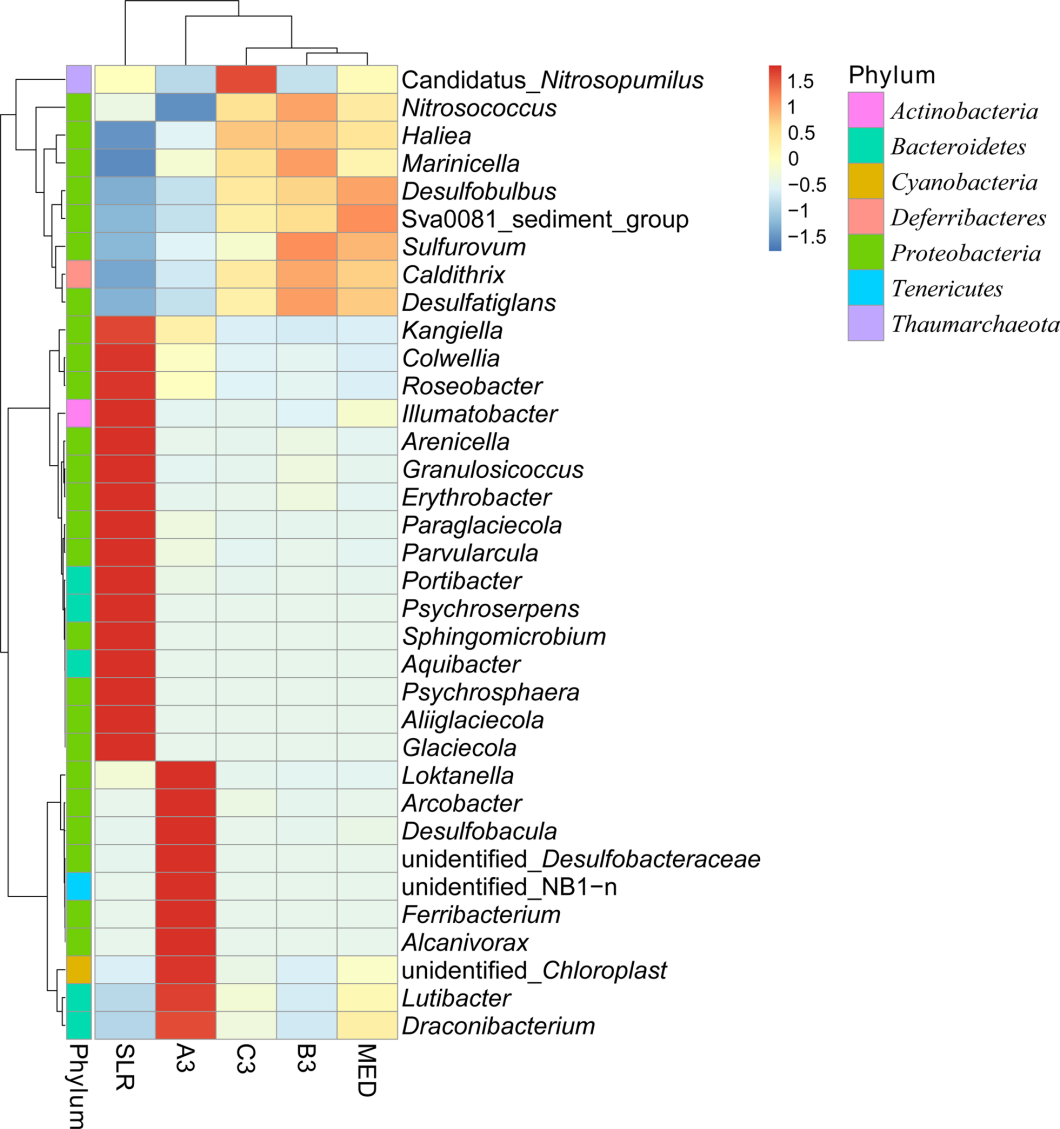
The heatmap analysis of the sediments in the direction perpendicular to the shoreline. The analyses were generated from the abundance data at the genus level. The top 35 genera were selected. The symbol A3 (B3, C3): the sampling site near the leak point; MED: control site in Jiaozhou Bay; SLR: control site outside of Jiaozhou Bay

The genera found at the greatest abundances in the five sites were studied by cluster analysis, shown as a heatmap in Fig. 6. The diversity at site A3 was completely different from the diversity at the other four sites. The main genera at site A3 were oil-degrading or PAH-degrading bacteria, such as *Alcanivorax* and *Lutibacter*. The community at site SLR also presented a distinct structure compared with the genera of the other four sites. The results of principal component analysis (PCA) indicated that bacteria at sites A3, B3, and SLR were represented by three types of communities (Additional file 1: Figure S1). The communities at sites C3 and MED indicated a high similarity in community structure at the genus level. In addition, the concentration of total petroleum hydrocarbon at site A3 was the highest, and it gradually decreased from B3 to C3; additionally, the communities at these sites were distinguished (clustered) following the distribution of pollutants. This was also demonstrated by the PCA results (Additional file 1: Figure S1).

### The biotoxicity of sediments with different levels of pollution

**Fig. 7:**
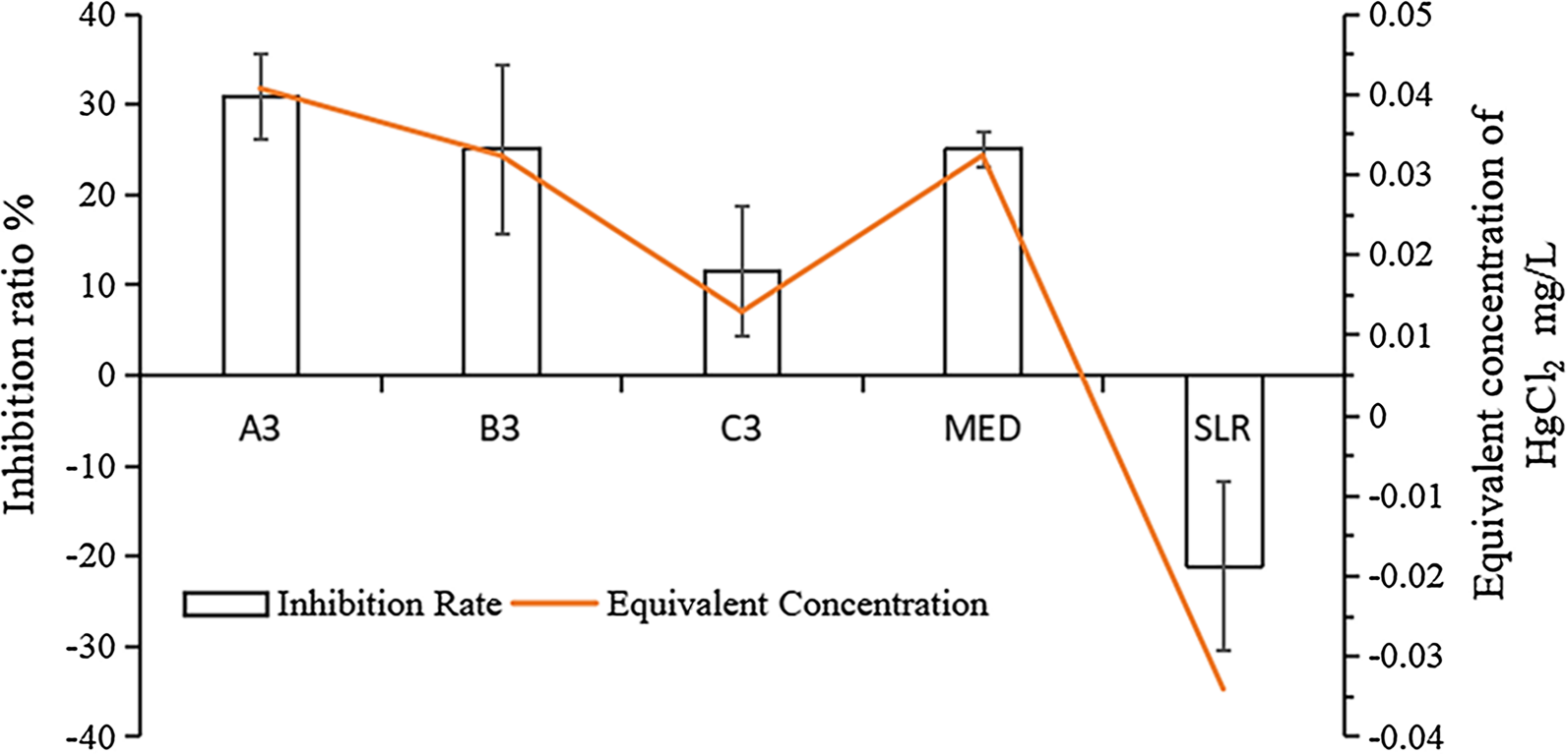
The luminous inhibition ratio and the equivalent concentration of HgCl_2_ in different sediments. The inhibition rates and equivalent concentrations of HgCl_2_ were shown in the figure. The inhibition rates were calculated by the negative control. The equivalent concentrations of HgCl_2_ were calculated according to the standard curve. The symbol A3 (B3, C3): the sampling site near the leak point; MED: control site in Jiaozhou Bay; SLR: control site outside of Jiaozhou Bay

The biotoxicity of sites A3, B3, C3, MED and SLR was evaluated by the luminescent bacteria method. The biotoxicity was presented as the equivalent concentration of HgCl_2_, which was calculated by the standard curve (Additional file 1: Figure S2). The sediment with the highest toxicity was collected from site A3, where the equivalent concentration of HgCl_2_ was 0.04 mg/L. The equivalent concentrations in the sediments from sites C3 and SLR were very low, no more than 0.01 mg/L (Fig. 7). To simplify the results of our data, toxicity was divided into three levels: a: no toxicity with an equivalent concentration below zero; b: low toxicity with an equivalent concentration ≤ 0.02 mg/L; and c: moderate toxicity with an equivalent concentration > 0.02 and ≤ 0.1 mg/L. Sediment from site SLR exhibited no toxicity; samples from site C3 showed low toxicity; and sediments from sites A3, B3 and MED exhibited moderate toxicity.

## Discussion

Offshore oil well blowouts or pipeline ruptures cause large amounts of oil to spill into marine environments, such as the Deepwater Horizon oil spill, the Bohai 19-3 oil spill and the Huangdao 11.22 oil pipeline explosion. Oil dispersants were widely applied in the marine oil removal process (Kleindienst et al. 2015). The dispersants lower the oil–water interfacial tension and break oil slicks into fine droplets. These dispersed oils may aggregate with suspended particulate material (SPM), such as clay minerals or organic matter, to form oil-SPM aggregates (OSAs) that get trapped on the bottom substrates in nearshore waters (Gong et al. 2014). It was estimated that 65% of released oil might form OSAs. This may result in serious pollution to the benthic environment because of the large amount of the spilled oil associated with each event. Studies have shown that oil including relatively labile low-molecular-weight n-alkanes, aromatics and BTEX (benzene, toluene, ethylbenzene, and *p*-, *m*-, and *o*-xylenes) remained in sediments 1 year after a spill (Liu et al. 2012). In our study, TPH was detected in all sediments from the nine sampling sites in Jiaozhou Bay. Five of the nine sites were moderately polluted according to Massoud’s et al. standard (1996). The TPH concentration of site A3 (near the oil leaking point) was close to a heavily polluted value (i.e., > 200 μg/g). In a previous study of Jiaozhou Bay, the concentrations of PAHs ranged from 1242.21 to 29,558.13 ng/g in sediments (Xue 2009). In the present study, the concentrations of PAHs were within this range at most sites. However, the concentration at site A3 was much higher than the average value found in Jiaozhou Bay. This means there was an exogenous input of PAHs to this site. On the other hand, the previous studies showed that the PAHs were mainly composed of high-molecular-weight components. In our study, low-ring PAHs accounted for a large proportion. Another study in Daya Bay showed that the increase in naphthalene content may be due to oil pollution, and this supports that the contamination of the sediments in Jiaozhou Bay was from crude oil (Sun et al. 2016).

The quick responses and succession of microbial communities in seawater or beach sediments were frequently studied after an oil spill (Hazen et al. 2010; Mason et al. 2012; Redmond and Valentine 2012). However, the impacts of the spilled oil on a sedimentary ecosystem after years of contamination have been less studied. Due to the low weathering effect, the oil might have a much longer impact time on the bacterial community. In our study, the bacterial communities at the five sites vertically distributed along the shoreline were considerably different. The bacterial communities were dominated by oil-degrading bacteria in seriously polluted sites. The abundance of *Firmicutes*, *Tenericutes* and *Cyanobacteria* accounted for 11.58% of the community at site A3, and species in these phyla were connected to oil contamination in marine environments (Ding et al. 2015; Ichor et al. 2016; Liu et al. 2012). *Cyanobacteria* showed high oil-degrading abilities and was considered to have good application potential in oil clean-up efforts (Raghukumar et al. 2001). *Firmicutes* were known to degrade hydrocarbons and were representative of the oil contamination at site A3. Additionally, they were the dominant phylum found in oil mousses (Liu and Liu 2013). Koo et al. (2015) found *Firmicutes* were a late responder after the oil contamination but had not been detected in the control. *Tenericutes* were also a late abundant phylum studied by Ding et al. (2015). Additionally, these two phyla were the main strains in the oil-polluted site A3, which meant that the oil pollution in the marine sediments at site A3 might last for years. The bacterial communities at the five sites were clustered by genera data. Site A3, with the highest concentrations of TPH and PAHs, was dominated by oil-degrading bacteria, such as *Lutibacter* and *Alcanivorax*, which was significantly different from the communities of the slightly polluted area and the non-polluted area. Thus, the results of the bacterial cluster could reflect the degree of oil pollution in sediments. When comparing between the moderately polluted and the non-polluted areas, *Erythrobacter* showed a big difference. From the studies of the Deepwater Horizon spill, *Erythrobacter* was the dominant genus in the oil mousses (Liu and Liu 2013). However, in this study, it was also one of the dominant genera found in the non-contaminated site SLR. The *Erythrobacter* spp. belonged to the aerobic anoxygenic phototrophic bacteria, which play a critical role in the carbon cycle in the ocean and these bacteria could metabolise organic substrates (Kolber et al. 2001; Yurkov and Beatty 1998). Why was the genus *Erythrobacter* more abundant in the clean area than in the polluted area? This might be because *Erythrobacter* spp. grow well in environments with high levels of organic matter content, such as oil or other types. The presence of *Erythrobacter* as dominant bacteria at the control site was probably due to the abundance of other forms of organic substrates.

In summary, the oil content in the water and along the coast quickly decreased because of the usage of dispersants and the weathering effect. In contrast, the oil in benthic sediments was more stable and difficult to degrade. Therefore, the spilled oil in sediments caused long-term impacts on the microbial community. We studied the oil polluted sites and two non-polluted control sites. The results showed that the microbial community abundance was consistent with the oil contamination which was supported by heatmap and PCA results. The sediments from seriously polluted, moderately polluted, slightly polluted, and non-polluted sites exhibited different microbial communities and biotoxicities. These differences to a certain extent could be used to evaluate the level of oil pollution in marine sediments. Understanding the impacts of spilled oil on indigenous microbial communities and identification of oil-degrading microbial groups were prerequisites for the clean-up efforts of oil-contaminated marine benthic ecosystems.

## Additional file

**Additional file 1: Figure S1.** PCA results of the sampling sediments. **Figure S2.** The standard curve of the biotoxicity analysis.

## Abbreviations

TPH: total petroleum hydrocarbons
PAHs: polycyclic aromatic hydrocarbons
CTAB: cetyltrimethyl ammonium bromide
SDS: sodium dodecyl sulphate
OTUs: operational taxonomic units
SPM: suspended particulate material
OSAs: oil-SPM aggregates
PCA: principal component analysis
