# Supplementary material for: Microbial diversity and ecotoxicity of sediments 3 years after the Jiaozhou Bay oil spill
Source: AMB Express. 2018 May 9;8:79. doi: 10.1186/s13568-018-0603-6 (PMC5943202; doi:10.1186/s13568-018-0603-6)
Supplement: Supplementary file 1 — Additional file 1: Figure S1. PCA results of the sampling sediments. Figure S2. The standard curve of the biotoxicity analysis. [file 13568_2018_603_MOESM1_ESM.doc]

**The microbial diversity and ecotoxicity in the sediments after three years of the Jiaozhou Bay oil spill**

Wei Gao ^a, b‡^, Xiaofei Yin ^b‡^, Tiezhu Mi^c, d, e^, Yiran Zhang ^b^, Faxiang Lin ^b^, Bin Han ^b^, Xilong Zhao ^b^, Xiao Luan ^b^, Zhisong Cui ^b^, Li Zheng ^b, e*^

^a^ College of Marine life, Ocean University of China, Qingdao, 266000, China;

^b^ Key laboratory for Marine bioactive substances and modern analytical Technology, First Institute of Oceanography, State Oceanic Administration of China, Qingdao, 266000, China;

^c^ College of Environmental Science and Engineering, Ocean University of China, Qingdao 266100, China;

^d^ Key Laboratory of Marine Chemical Theory and Technology, Ministry of Education, Qingdao 266100, China;

^e^ Laboratory for Marine Ecology and Environmental Science, Qingdao National Laboratory for Marine Science and Technology, Qingdao 266071, China.

^*^Corresponding author

Mailing Address: the First Institute of Oceanography, State Oceanic Administration, No. 6 Xianxialing Road, Qingdao, Shandong Province, 266061, PR China

Phone: (86) 532-88961802.

Fax: (86) 532 88963253.

E-mail: [zhengli@fio.org.cn](mailto:zhengli@fio.org.cn)

**Additional file**


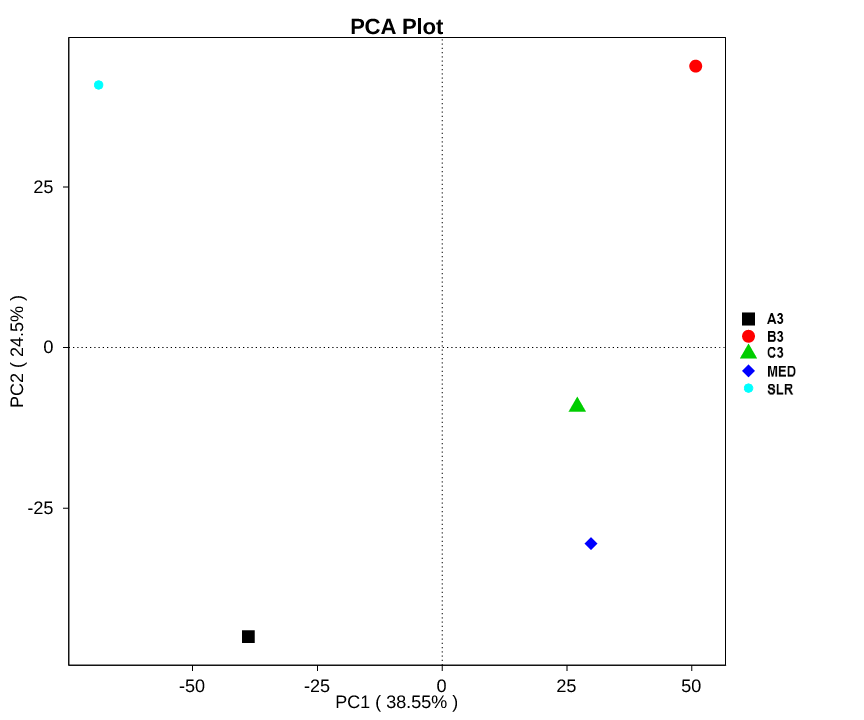


Figure S1 PCA results of the sampling sediments

The symbol A3 (B3, C3): the sampling site near the leaking point; MED: control site in Jiao Zhou bay; SLR: control site out of Jiao Zhou bay.





Figure S2 The standard curve of the biotoxicity analysis

HgCl_2_ was used as a reference standard toxicant and the R^2^ value was 0.93.
